# Discovery of Amorphous Iron Hydrides via Novel Quiescent Reaction in Aqueous Solution

**DOI:** 10.1038/s41598-020-63124-2

**Published:** 2020-04-10

**Authors:** Kohei Taguchi, Kazuteru Shinozaki, Hideyuki Okumura, Chishiro Michioka, Kazuyoshi Yoshimura, Keiichi N. Ishihara

**Affiliations:** 1 Department of Material Science, Graduate School of Nanobioscience, Yokohama City University, 22-2 Seto, Yokohama, 236-0027 Japan; 2 Department of Socio-Environmental Energy Science, Graduate School of Energy Science, Kyoto University, Yoshidahonmachi, Sakyo-ku, Kyoto, 606-8501 Japan; 3 Department of Chemistry, Graduate School of Science, Kyoto University, Kitashirakawa Oiwake-cho, Sakyo-ku, Kyoto, 606-8502 Japan

**Keywords:** Metals and alloys, Synthesis and processing

## Abstract

Novel amorphous iron hydrides (AIHs) are synthesized for the first time under ambient conditions by employing novel “quiescent reaction”, without stirring for mixing solutions, during a conventional aqueous reduction-precipitation process. The kind and morphology of AIHs are dependent on the processing condition, where two types are found, with one form consisting of a tangle of uniform nanowires and the other being granular in nature. Both AIHs undergo transformation to crystalline α-Fe by heat treatment at 600 °C. The nanowire AIH exhibits the hydrogen content of 0.10 wt%, while the granular AIH of 0.22 wt%. Their magnetic and thermal properties are accordingly different, and the non-diffusive hydrogen contributes to stability of AIHs. It is strongly suggested that, by use of quiescent reaction, iron-hydrogen clusters are formed and preserved at an early stage of precipitation reaction, and subsequently aggregated into novel AIHs, preventing α-Fe crystallization. Hence, the AIHs would be categorized as metastable hydrides stabilized with iron-hydrogen clusters. In addition, newly discovered quiescent reaction in aqueous solution, from which unprecedented AIHs are derived, sheds new light on fundamental and essential aqueous reaction.

## Introduction

Hydrogen is considered to be attractive as a clean and sustainable fuel in the near future^1^. In order to realize the so-called hydrogen economy, a lot of investigation has been conducted for hydrogen production and storage, especially on materials^1–6^, where one of the key phenomena is interaction between metal and hydrogen. In this light, not only metallic hydrides^1^ but also amorphous alloys are interesting research topics as often exhibiting their larger hydrogen absorption capacity compared with counterpart crystalline alloys^5^. Further, amorphization is reported to be induced by hydrogenation in solid state reactions for some crystalline alloys^7^. Many metallic elements such as Li, Na, K, Mg, Ca, Al, Ti, Ni, and Zr, are investigated to apply for hydrogen storage materials^1,5^. However, though earth-abundant, iron has hardly been considered as the candidate, because of its poor interaction with hydrogen. It is known that solubility of hydrogen in iron under ambient conditions is extremely low because of large positive heat of solution^8^. Iron hydrides thus far found to be stable are only at hydrogen pressures in a gigapascal range^9,10^. Recently, it was also reported that amorphous iron (II) hydride (FeH_2_) could be synthesized under a pressure of 100 bar via hydrogenolysis of an iron metal complex in solution^11^. Notwithstanding this, synthesis of iron hydrides under ambient conditions has not been reported.

In the present study, we report a discovery of amorphous iron hydrides (AIHs), which is synthesized via a novel modified method of a reduction-precipitation process under ambient conditions. The newly-produced AIHs are stable under ambient conditions, and transform into pure α-Fe upon annealing. Two types of AIHs, each with different non-diffusive hydrogen concentrations, are found; one with granular forms and the other a tangle of nanowires^12^.

In terms of a structural point of view, a glassy state of elemental pure iron is not easily formed^13–15^ without employing an alloying technique. Addition of eutectic alloy-forming elements such as B or P, for example, is effective for producing amorphous iron alloys that are stable under ambient conditions^16^. However, upon heating, the amorphous alloys would transform into various crystalline compound phases other than pure crystalline iron. By taking advantage of nanocrystallization of pure amorphous iron, AIHs may serve as building blocks in manufacturing nanostructure-controlled iron parts, such as 3D printing, upon subsequent heat treatment^12^. Although iron nanoparticles and nanowires could be produced under ambient conditions via similar reduction-precipitation processes using aqueous solutions with Fe^2+^ ions and reducing agents, the nanoparticles produced as such would be generally comprised of crystalline iron^17,18^. Our devised method does not disturb the nascent early structure (as in embryo development) in a solution and its subsequent growth during the precipitation process. Consequently, AIHs are realized under ambient conditions for the first time.

Employing our novel approach would permit one to eliminate an effect of non-essential variables on the reaction progress. This may enable to further determine the key phenomena involved in the reaction, shedding light on the mechanism. In this paper, the devised synthetic methods are presented in detail, and characteristic features of AIHs are described. A possible formation mechanism is discussed as well.

## Experimental section

### Synthesis

#### (1) Drop-by-drop mixing method

A simple yet ingenious method was used to achieve novel “quiescent reaction” during precipitation process. Specific and unique methodological feature was the significantly-slow (drop-by-drop) addition of aqueous NaBH_4_ solution to Fe^2+^ solution, without stirring and without addition of any other chemicals. Gentle and almost-quiescent conditions, referred to as “quiescent reactive (QR) conditions”, were used for mixing.

Aqueous solutions of 0.067 mol/L Fe^2+^ and 0.063 mol/L NaBH_4_ were used, as it had been reported that concentrations should be in the ranges 0.015-0.15 mol/L and 0.015-1.0 mol/L, respectively, or otherwise, iron oxides, hydroxides, or borides would be mostly formed (Fig. 61 in ref. ^12^). The NaBH_4_ solution in distilled water (25 mL) was added slowly, dropwise without stirring at a rate of 5 mL/min, to aqueous FeSO_4_ solution (16 mL) in a 100 mL beaker. The beaker was placed on a neodymium magnet. With addition of each drop, the mixture became cloudy due to formation of small black precipitates, along with many bubbles, and the precipitates drifted to the bottom of the beaker due to magnetic attraction. After the mixture was left to stand for 5 min, the remaining solution was decanted off. Precipitated solids attached at the bottom of the beaker were rinsed with water/ethanol, and dried at 150 °C for 2 min in a glass tube that was evacuated by a rotary pump.

#### (2) Pour-and-stir mixing method

An aqueous solution of NaBH_4_ (75 mL, 0.063 mol/L) was slowly poured into an aqueous solution of FeSO_4_ (48 mL, 0.067 mol/L) in a 200 mL beaker at a rate of 4 mL/sec, and the mixture was stirred for 10 min using a glass rod. A large number of bubbles, which were produced during reduction of Fe^2+^ ions by NaBH_4_, aided the stirring process. Precipitated material was filtered, rinsed with water/ethanol, and dried in a desiccator at room temperature.

#### (3) Dilute Fe^2+^ solution method

The dilute NaBH_4_ solution (60 mL, 0.026 mol/L) was added, using likewise drop-by-drop procedure in (1), to dilute FeSO_4_ solution (120 mL, 0.0027 mol/L) in a 200 mL beaker at a rate of 10 mL/min (under QR conditions). Here, the ethanol-water mixture (25 wt% ethanol) was used as a solvent, and the beaker was not placed on a magnet. Black precipitates appeared with bubbles during the addition. The mixture was then left to stand for 15 min, and the black solid was collected on the bottom of the beaker using a magnet. After decanting the remaining solution, the solid was rinsed with water/ethanol. After degassing at 200 °C for 2 min in a glass tube that was evacuated by a rotary pump, sample material was obtained. Several repetitions of above procedure resulted in more material, which was characterized as outlined below.

### Characterization

Various analytical techniques were employed to characterize the samples: SEM (scanning electron microscopy) with secondary electron emission using a KEYENCE VE-9800 electron microscope, TEM (transmission electron microscopy) with selected area electron diffraction (SAED) in a JEOL JEM-2010, XRD (X-ray diffractometry) with Cu Kα radiation in the range 2θ = 20–120° in a Bruker AXS NEW D8 ADVANCE diffractometer, SQUID (superconducting quantum interference device) magnetometry in a Quantum Design MPMS-XL system for measurement of magnetic hysteresis and temperature-dependent magnetization, and DSC (differential scanning calorimetry) in a Shimadzu DSC-60 calorimeter using an Al pan under a N_2_ atmosphere with a heating rate of 3 °C/min. Non-diffusive hydrogen content of a sample, after degassing at 200 °C for 2 min in a glass tube evacuated with a rotary pump to remove free hydrogen^19^, was determined using a HORIBA EMGA-621A, based on the JIS (Japanese Industrial Standards) Z 2614, “General Rules for Determination of Hydrogen in Metallic Materials,” and JIS H 1619 “Titanium and Titanium Alloys – Determination of Hydrogen Content.” Briefly, the sample, around 100 mg or less, was melted by heating in an Ar gas flow to extract hydrogen gas; the hydrogen gas was separated using a separation column for measurement of thermal conductivity, which was then converted into amounts of hydrogen by referring to the calibration curve previously provided^12^.

## Results

**Figure 1:**
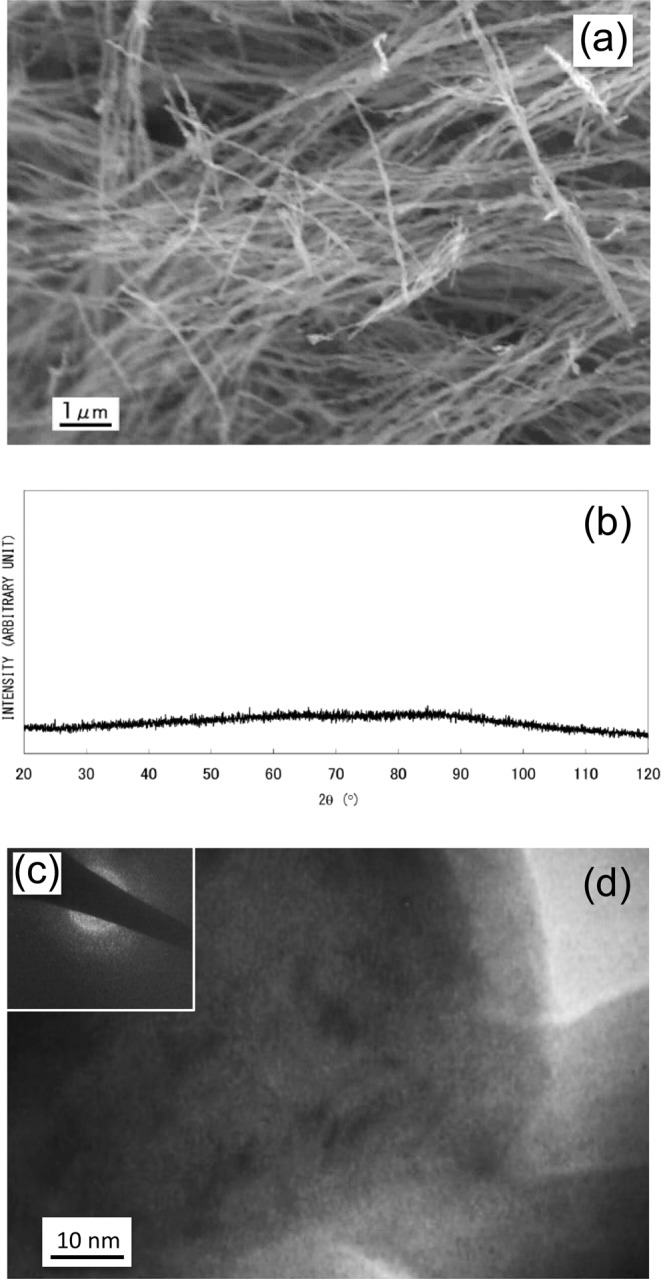
(**a**) Scanning electron microscopy image of nanowires (AIH-1) obtained by the drop-by-drop mixing method^12^, (**b**) X-ray diffraction analysis^12^, (**c**) selected-area electron diffraction (SAED) pattern, and (**d**) transmission electron microscopy (TEM) image. The SAED pattern was obtained from an area larger than the TEM image.

**Figure 2:**
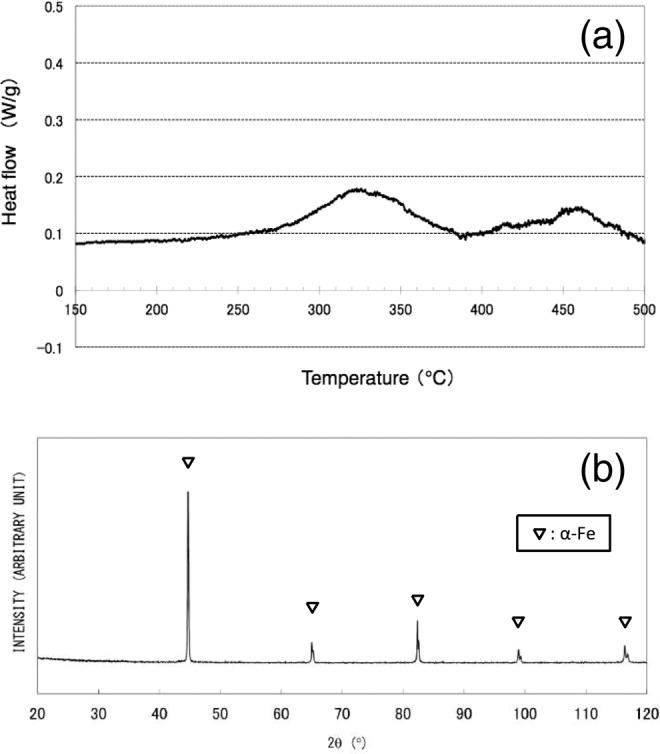
(**a**) Differential scanning calorimetry results for nanowires (AIH-1)^12^. (**b**) X-ray diffraction analysis for AIH-1 after heat treatment at 600 °C^12^.

An SEM image of nanowires obtained by drop-by-drop mixing (Synthesis (1)) is shown in Fig. 1(a). It is revealed that nanowire morphology is relatively uniform but tangled, and exhibits a diameter of around 100 nm with a length of at least 10 μm. The nanowire material is considered to exhibit an amorphous phase or a very fine crystalline phase, because no distinctive peaks appear in the XRD pattern (Fig. 1(b)), and only diffuse rings appear in the SAED pattern (Fig. 1(c)). The TEM micrograph shown in Fig. 1(d) reveals that the nanowire contains sub-structures with dimensions of less than 10 nm, indicative of clustered regions. As magnified SEM observation and TEM analyses indicate periodic bulges on the sides of nanowires, it is conjectured that they are formed via a self-assembly mechanism: Nanoparticles with uniform diameters of around 100 nm are produced, and almost simultaneously connected with each other along directions of applied magnetic field lines^12^. Even in amorphous phase, existence of fundamental magnetic unit is suggested to exhibit such anisotropy in morphology under magnetic field. Non-diffusive hydrogen content remained after degassing at 200 °C for 2 min was determined to be 0.10 wt%. A DSC analysis gave two broad exothermic peaks at around 320 °C and 460 °C, as shown in Fig. 2(a), suggesting a stepwise transformation. When the sample was subsequently heated at 600 °C for 60 min in a sealed glass tube (that was evacuated to prevent oxidation), sharp peaks associated with a single α-Fe phase appeared in the XRD pattern, as shown in Fig. 2(b). According to previous work^12^, when iron compounds such as iron oxides, hydroxides, or borides, are present in precipitated material, they can be detected through XRD analysis. The present results indicate that obtained amorphous (or amorphous–like) material consists of iron and non-diffusive hydrogen, and is categorized as AIH-1. Molar ratio of iron to hydrogen is estimated to be *n*_Fe_:*n*_H_ ≈ 20: 1.1.

**Figure 3:**
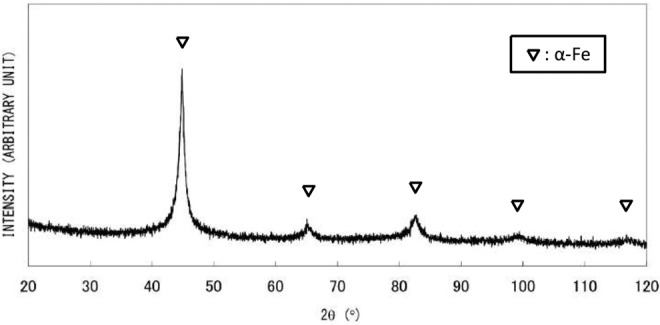
X-ray diffraction analysis of material produced by the pour-and-stir mixing method^12^.

For comparison purposes, a pour-and-stir mixing method, as mentioned in synthesis (2), was also employed to precipitate iron material, and after drying at room temperature the broad peaks of α-Fe were observed in the XRD pattern, as shown in Fig. 3. Therefore, slow and gentle addition via drop-by-drop mixing (QR conditions), which achieves quiescent reaction, is critical for preparing AIH-1.

**Figure 4:**
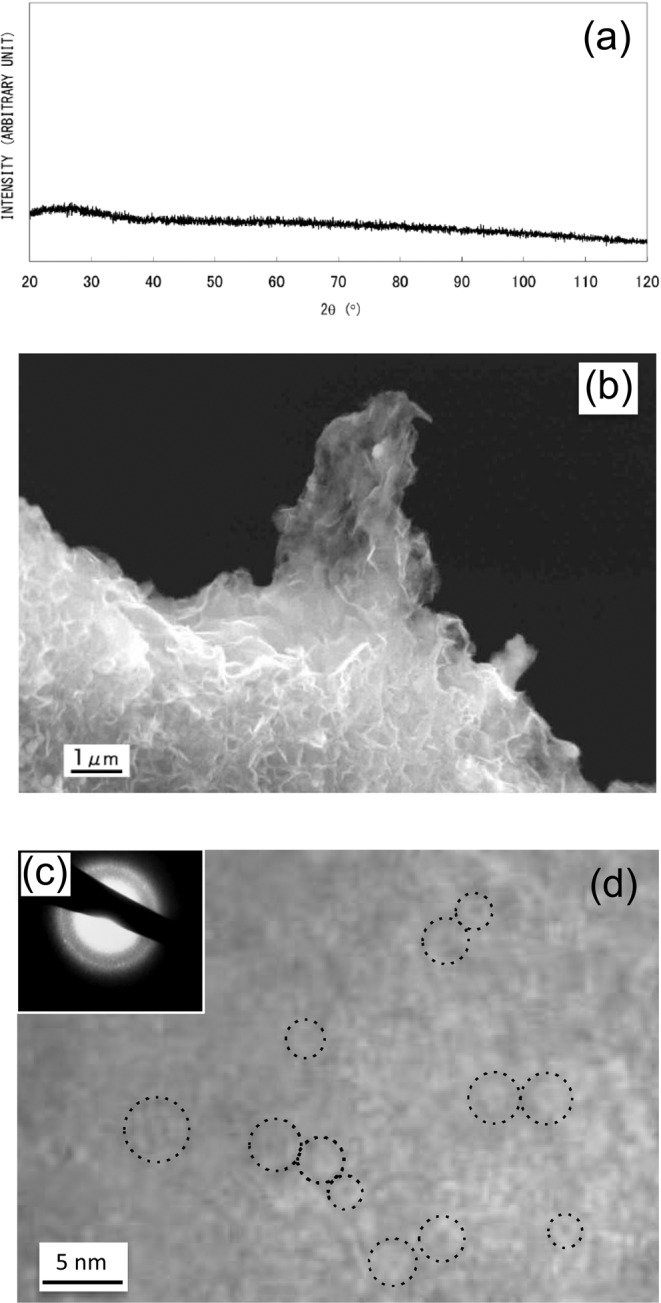
(**a**) X-ray diffraction analysis of granular material (AIH-2)^12^, (**b**) scanning electron microscopy image of a fracture surface of a grain^12^, (**c**) selected area electron diffraction (SAED) pattern, and (**d**) transmission electron microscope (TEM) image, where dotted circles indicate clusters or regions with medium-range order. The SAED pattern was obtained from an area larger than the TEM image.

**Figure 5:**
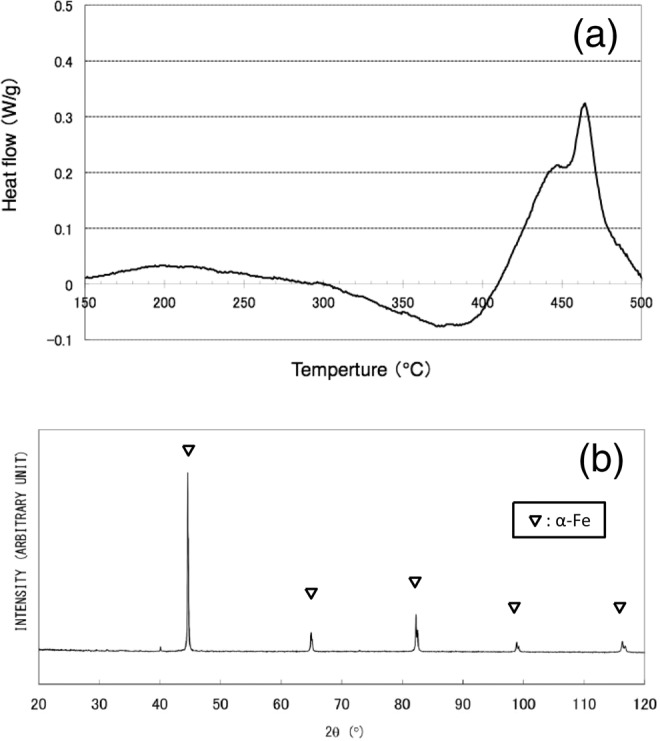
(**a**) Differential scanning calorimetry for granular material (AIH-2)^12^. (**b**) X-ray diffraction analysis for AIH-2 after heat treatment at 600 °C^12^. The small peak at ca. 40 degree is unknown.

In the case of the dilute Fe^2+^ solution method (synthesis (3)), which employs ethanol-water solvent and drop-by-drop mixing under QR conditions, the obtained material exhibits no distinctive XRD peaks (Fig. 4(a)). Fig. 4(b) shows an SEM image of fractured surface of a granule with a diameter of around 300 μm. The irregular surface morphology is typical of an isotropic amorphous structure. The sample morphology is quite different from AIH-1 nanowires (Fig. 1(a)). The SAED pattern obtained from granules exhibits diffuse rings, as shown in Fig. 4(c), indicating an amorphous phase or a very fine crystalline structure, which is consistent with XRD results. The TEM micrograph in Fig. 4(d) reveals sub-structures including some clustering or regions with medium-range order (MRO) with dimensions of less than 5 nm. Non-diffusive hydrogen content was determined to be 0.22 wt%, almost twice as large as that of AIH-1. The DSC analysis showed a large, broad endothermic peak and one or two exothermic peaks (Fig. 5(a)), which may be attributed to glass transition (structural relaxation) and crystallization, respectively; the DSC results are quite different from those for AIH-1 (Fig. 2(a)). Upon heating the granules at 600 °C for 60 min under deoxygenated conditions, sharp XRD peaks of a pure α-Fe crystalline phase are obtained, as shown in Fig. 5(b). Therefore, obtained amorphous (or amorphous-like) material can be categorized as AIH-2, with a ratio of *n*_Fe_:*n*_H_ ≈ 8:1.

**Figure 6:**
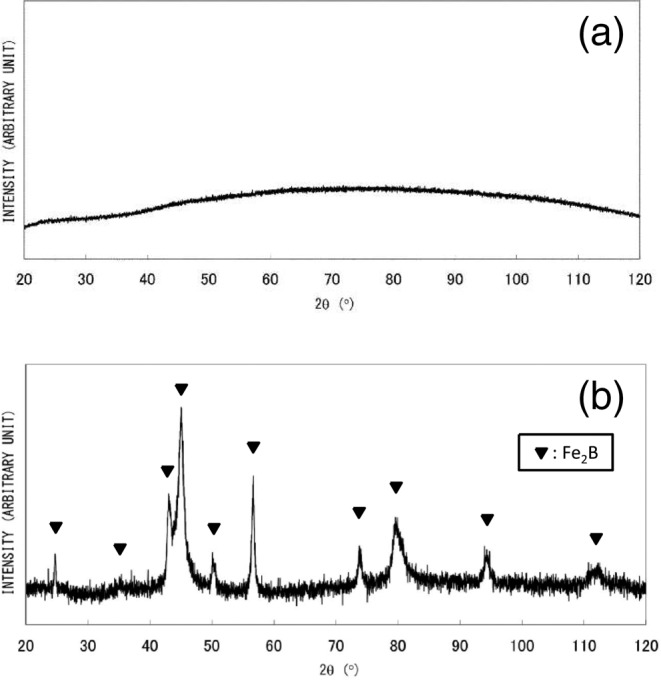
X-ray diffraction analysis of material produced by the dilute Fe^2+^ solution method without ethanol: (**a**) without heat treatment and (**b**) after heat treatment at 600 °C^12^.

In the dilute Fe^2+^ solution method (synthesis (3)), the presence of ethanol in a solvent is extremely important. Although XRD analysis of formed precipitates exhibits no distinctive peaks (Fig. 6(a)) even without usage of ethanol, broad peaks of Fe_2_B are observed upon heat treatment at 600 °C (Fig. 6(b)). Thus, addition of ethanol to a water solvent plays a decisive role in the synthesis of AIH-2, preventing formation of iron borides.

**Figure 7:**
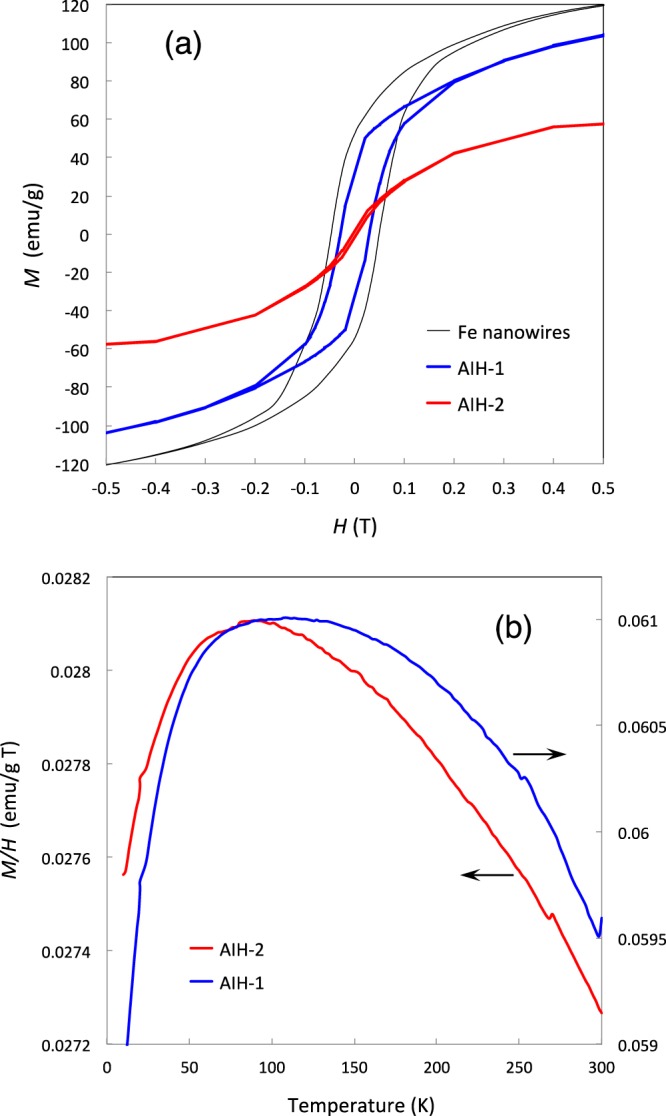
(**a**) Magnetic hysteresis loops measured at 300 K for AIH-1 and AIH-2, shown by the blue and red lines, respectively, where *M* is the magnetization and *H* is the magnetic flux density. The black line is for α-Fe nanowires with a 40 nm diameter^20^. (**b**) Temperature dependence of *M/H* for AIH-1 and AIH-2 under *H* = 0.1 T, shown by blue and red lines, respectively.

Magnetic hysteresis loops (M-H loops) for AIH-1 and AIH-2 are quite different, as shown in Fig. 7(a). The former exhibits larger hysteresis than the latter, with higher magnetization and coercivity as well. The results indicate that magnetization of AIHs decreases with an increase in non-diffusive hydrogen concentration due to an interaction between iron and hydrogen. Higher coercivity of AIH-1 nanowire may derive from its shape anisotropy^20^. Fig. 7(b) shows T(temperature)-dependence of normalized magnetization (M/H) for both AIHs. A broad peak is exhibited for each profile, where the peak temperature for AIH-2 (90 K) is slightly lower than AIH-1 (110 K). Magnetization for AIH-2 is less than half that for AIH-1 at each temperature, consistent with hydrogen content: 0.10 wt% (AIH-1), 0.22 wt% (AIH-2).

Magnetic properties described above may well correspond to different but uniform morphologies of AIH-1 and AIH-2. In the case of AIH-1, nanowires exhibit a uniform diameter of about 100 nm (Fig. 1(a)), while granular AIH-2 material exhibits a poorly-defined isotropic structure (Fig. 4(b)) with MROs or clustered regions less than 5 nm (Fig. 4(d)). Distinct morphologies suggest difference in fundamental magnetic units, depending on hydrogen content.

## Discussion

By employing novel “quiescent reaction” during aqueous reduction-precipitation procedure, we have selectively synthesized novel phases “AIH-1” and “AIH-2”, through controlling (i) mixing method, (ii) Fe^2+^ concentration, and (iii) solvent. Both AIHs are considered as hydrogen-stabilized amorphous iron, based on the following: (1) AIHs contain non-diffusive hydrogen at specific concentrations, (2) They are stable under ambient conditions and transform into crystalline α-Fe when heated, (3) Thermal stability of AIHs is much higher than that previously reported for amorphous iron produced by ultrasound^21^, where the crystallization temperature is 460 °C for the former and 308 °C for the latter, (4) They exhibit distinct characteristics in terms of DSC analysis, magnetic property, and morphology, corresponding to non-diffusive hydrogen concentration. These findings strongly suggest that AIHs are not solid solutions (of metallic iron with hydrogen) but compounds with non-diffusive hydrogen stabilized via electrostatic attraction or chemical bonding. Moreover, since crystalline phases are not detected, they would exist as amorphous (or amorphous-like) phases, which crystallize into bcc α-Fe when heated. For these reasons, AIHs are categorized as metastable hydrides without long-range order.

The DSC analysis shown in Figs. 2(a) and 5(a) exhibits a possible difference in phase transformation into α-Fe crystalline phase from AIH-1 and AIH-2, which is related to hydrogen release and crystallization. Two exothermic reactions are indicated for AIH-1, with the first peak at around 320 °C and the second at around 460 °C, which may correspond to different kinds of iron-hydrogen bonds involved in AIH-1. On the other hand, very-close two exothermic peaks can be seen in Fig. 5(a) at around 450 °C for AIH-2, suggesting two successive exothermic reactions. Although the transformation pathway is unknown, both AIHs may exhibit common ground in that the Fe-H bonding is broken for hydrogen release prior to α-Fe crystallization. From the consideration, it is suggested that the difference of DSC transformation information between Figs. 2(a) and 5(a) corresponds to their amorphous structures related to iron-hydrogen bonds.

Plausible formation mechanisms of AIHs include: (1) Fe(0) aggregates are first precipitated, followed by their absorption of hydrogen to form AIHs; (2) Fe-H bonds are first formed, followed by attachment of Fe(0) and/or detachment of H to form AIHs. The former mechanism is analogous to that for Zr-based alloys^22^, where hydrogen is more readily absorbed in amorphous alloys than in crystalline alloys, because absorbed hydrogen atoms preferentially occupy tetrahedral, hexahedral and/or octahedral sites with topological distortion and chemical fluctuation. The latter mechanism can be inferred, given that FeH_2_ phase is reported to form by oxidative addition of H_2_ to iron complex in toluene under 100 bar of gaseous hydrogen^11^. Taking advantage of solution processes, Fe-H bonds can be created at significantly lower hydrogen pressures, compared with the gigapascal range reported for solid processes^10^.

Our study clearly shows the so-called QR conditions are indispensable for AIHs formation. Otherwise, as in the case of pour-and-stir mixing method (synthesis (2)), crystalline bcc α-Fe would be formed. Given the metastability of once-formed AIHs, a “precursor” must be kept under QR conditions to prevent an incipient AIH embryo from crystallizing into bcc α-Fe at an early stage of precipitation reaction. Under QR conditions, AIHs formation would essentially have priority over crystallization. Thus, our simple-yet-devised drop-by-drop method under QR conditions would be ideal to obtain the prior products, AIHs, in contrast to stirring conditions that are normally employed for reduction-precipitation reactions.

Solid metallic iron is known not to react with hydrogen under ambient conditions^11^, while hydrogen adsorption by small iron clusters is predicted to occur based on calculations^8^. Since hydrogen would participate in and contribute to stabilization of AIHs, it is suggested that small nascent aggregates consisting of Fe(0), formed by reduction of Fe^2+^, adsorb hydrogen to form special precursors under QR conditions, whereby AIHs are formed. In the case of AIH-2 synthesis (synthesis (3)), the role of ethanol is to increase hydrogen concentration during the reaction^23^. It is then proposed that another precursor different from that for AIH-1 is produced in the presence of higher hydrogen concentration of diluted Fe^2+^ solution under QR conditions, whereby AIH-2 is formed.

If the AIH species contains crystalline structure, the crystallite size would be extremely small, given that no distinctive peaks are detected in the XRD patterns^24^, consistent with our TEM analyses. For an ideal, smallest iron cluster (tetrahedral (T_d_) structure: Fe_4_) with hydrogen (Fe_4_H cluster), the formation heat would be exothermic when hydrogen is adsorbed on top of an iron tetrahedron^8^, despite that dissolution of hydrogen in iron is endothermic with a large positive enthalpy of solution^8^. It is then proposed that, according to synthesis conditions, different kinds of iron-hydrogen clusters, preserved as precursors under QR conditions, aggregate to form AIH-1 and AIH-2. It is suggested that specific precursors selectively synthesized are fundamental units, like molecules, to form AIHs and to manifest their distinguished characteristics, i.e. DSC profiles, magnet properties and morphology. It is reasonable to assume AIHs possess iron-hydrogen clusters, e.g. imperfect polyhedral forms^25,26^, that derive from incipient precursors without crystallizing to release hydrogen, so as to form amorphous hydrides. Further investigation is required for elucidating structures of precursors, clusters, and bulk-AIHs, in order to reveal hydrogenation mechanism of unprecedented metallic hydrides.

The results point to discovery of amorphous hydrides stabilized with iron-hydrogen clusters. On this basis, there is a possibility that formation heat calculation of other metal-hydrogen clusters will lead to realization of different cluster-stabilized hydrides involving other metallic elements. Finally, new comprehension on aqueous reaction gained from simple-yet-devised quiescent reaction, which leads to discovery of AIHs, sheds new light on fundamental chemical reaction in aqueous solution. In this context, new opportunities afforded by the quiescent reaction can be expected to impact on other reactions, leading to discovery of novel compounds.

## Conclusions

Novel amorphous iron hydrides (AIHs), previously unreported, are synthesized under ambient conditions via “quiescent reaction” during a conventional aqueous reduction-precipitation process. The quiescent reaction is newly discovered through gentle mixing of solutions, without stirring, in contrast to conventional mixing methods. In order to obtain AIHs, the quiescent reaction is indispensable, preventing crystallization into bcc α-Fe. It is speculated that, during quiescent reaction, “precursors”, i.e. specific iron-hydrogen clusters according to specific solutions, are formed and preserved at an early stage of precipitation, which are essential to their aggregating into AIHs. The AIHs exhibit distinct characteristics, plausibly corresponding to their fundamental units based on the precursors selectively synthesized. Thus, the AIHs are suggested to be cluster-stabilized amorphous metallic hydrides. The novel quiescent reaction that has been demonstrated to realize unprecedented AIHs formation, without iron crystallization, would provide a new insight on chemical reaction in solution.

## References

[1] Mandal TK, Gregory DH (2010). Hydrogen: A future energy vector for sustainable development. P. I. Mech. Eng. C.-J. Mec..

[2] Lee, H. *et al*. Tuning clathrate hydrates for hydrogen storage. *Nature* **434**, 743–746, 10.1038/nature03457 (2005). 10.1038/nature03457 15815624

[3] Chen, K. *et al*. Converting H^+^ from coordinated water into H^−^ enables super facile synthesis of LiBH_4_. *Green Chem.* **21**, 4380–4387, 10.1039/c9gc01897b (2019).

[4] Sun Y, Shen C, Lai Q, Liu W, Wang D–W, Aguey-Zinsou K–F (2018). Tailoring magnesium based materials for hydrogen storage through synthesis: Current state of the art. Energy Storage Materials.

[5] Eliaz N, Eliezer D (1999). An overview of hydrogen interaction with amorphous alloys. Advanced Performance Materials.

[6] Aguey-Zinsou K–F, Ares-Fernández J-R (2008). Synthesis of colloidal magnesium: A near room temperature store for hydrogen. Chem. Mater..

[7] Aoki K, Masumoto T (1993). Solid state amorphization of intermetallic compounds by hydrogenation. J. Alloys. Compd..

[8] Minot CH, Demangeat C (1987). The iron-hydrogen system: Lattice location of hydrogen, heat of formation, and hydrogen-hydrogen binding energy. J. Chem. Phys..

[9] Antonov, V. E. *et al*. High-pressure hydrides of iron and its alloys. *J. Phys.: Condens. Matter* **14**, 6427–6445, http://stacks.iop.org/JPhysCM/14/6427 (2002).

[10] Badding JV, Hemley RJ, Mao HK (1991). High-pressure chemistry of hydrogen in metals: *In situ* study of iron hydride. Science.

[11] Morris L, Trudeau ML, Lees MR, Hanna JV, Antonelli DM (2014). On the path to bulk FeH_2_: Synthesis and magnetic properties of amorphous iron (II) hydride. J.Alloy. Compd..

[12] Taguchi, K., Shinozaki, K. & Takayasu, S. Metal-based structure or nanoparticles containing hydrogen, and method for producing same. U.S. Patent 10125019, issued November 13, 2018 (U. S. patent application publication US 2016/0311685, published October 27, 2016) and Japanese patent 6050501, issued December 2, 2016.

[13] Notomi K, Yamakawa K, Fujita FE (1970). Recovery process of iron films deposited on low temperature substrate. J. Phys. Soc. Jpn..

[14] Kim Y-W, Lin H-M, Kelly TF (1988). Solidification structures in submicron spheres of iron-nickel: Experimental observations. Acta metall..

[15] Enomoto N, Hirata S, Inada M, Hayashi K (2017). Crystallization behavior of iron-based amorphous nanoparticles prepared sonochemically. Ultrason. Sonochem.

[16] Takahashi M, Koshimura M, Abuzuka T (1981). Phase diagram of amorphous and crystallized Fe-B alloy system. Jpn. J. Appl. Phys..

[17] Kawamori M, Yagi S, Matsubara E (2014). Iron alloying effect on formation of cobalt nanoparticles and nanowires via electroless deposition under a magnetic field. J. Electrochem. Soc..

[18] Kassaee, M. Z., Motamedi, E., Rahnemaie, R. & Mikhak, A. Nitrate elimination from water by Fe nanoparticles fabricated by two methods: Reduction vs. arc fabrication. Proceedings of the 3rd Conference on Nanostructures (NS2010) March 10-12, 2010, Kish Island, I.R. Iran.

[19] Takai K, Watanuki R (2003). Hydrogen in trapping states innocuous to environmental degradation of high-strength steels. ISIJ Int..

[20] Han R, Li W, Pan W, Zhu M, Zhou D, Li F-S (2014). 1D magnetic materials of Fe_3_O_4_ and Fe with high performance of microwave absorption fabricated by electrospinning method. Sci. Rep..

[21] Suslick KS, Choe S-B, Cichowlas AA, Grinstaff MW (1991). Sonochemical synthesis of amorphous iron. Nature.

[22] Suzuki K (1983). Structure and properties of amorphous metal hydrides. J.Less-Common Met.

[23] Purwanto, Deshpande RM, Chaudhari RV, Delmas H (1996). Solubility of hydrogen, carbon monoxide, and 1-octene in various solvents and solvent mixtures. J. Chem. Eng. Data.

[24] Patterson AL (1939). The Scherrer formula for X-ray particle size determination. Phys. Rev.

[25] Hirata, A. *et al*. Geometric frustration of icosahedron in metallic glasses. *Science* **341**, 376–379, 10.1126/science.1232450 (2013). 10.1126/science.1232450 23845945

[26] Fan, C. *et al*. Structural model for bulk amorphous alloys. *Appl. Phys. Lett.* **89**, 111905–111908, 10.1063/1.2345276 (2006).

